# Concentrated Foods

**Published:** 1899-06-24

**Authors:** 


### CONCENTRATED FOODS.

In the same lecture Dr. Dickinson dealt very summarily with many popular ideas in regard to the utility of concentrated foods. Food, he holds, cannot be concentrated. "For 'human nature's daily food' are required certain weights of nitrogen, carbon, oxygen, and hydrogen, which are ultimate forms of matter, and not capable of concentration or further reduction to essential principles. An ox," he says, "cannot be got into a teacup by any other process than by leaving the greater part of the animal outside. The stomach of the average man demands every twenty-fours 21 grammes of nitrogen, or about three-quarters of an ounce; 307 grammes of carbon, or about 11 ounces; 12 grammes of hydrogen, not quite half an ounce; besides sulphur and salts. This is required to make up for the waste—to restore what leaves his body in various shapes. Three-quarters of a pound of carbon cannot be got into less than three-quarters of a pound. The nutritive effect cannot be separated from the bulk. The man must have his pound of flesh . . . A drachm of Liebig's extract cannot supply more than a drachm. Quality cannot supply the place of quantity." Perhaps it would have been only fair to add that Liebig never said it did, and always maintained that the bulk was to be made up by the addition of other forms of food. Dr. Dickinson, however, states that although these concentrated extracts are not, properly speaking, foods, they have their special use as supplementary to the essentials of food, and as an example of this he says that he has long been in the habit of using them to help to make up for the loss of the salts by suppuration, and so prevent or retard the consequent lardacity. They are to be looked upon, he says, as additions to the essential diet, not substitutes for it. All of this is fairly obvious. That an ox cannot be got into a teacup is plain to everyone, but for all that there is a great deal in an ox that we do not eat, and when a food is concentrated in the sense of eliminating useless material from it, it still remains a true food. The fact is that such a discussion as this is to a large extent a play upon words. Sugar, for example, is a concentrated carbo-hydrate food, just as pure oil is a concentrated hydro-carbonaceous food, that is, in both cases they are concentrated by the removal from them of all extraneous matter, such as woody and animal fibres and such like presumably "indigestible" matters. On neither one nor the other of them, however—nor, indeed, on both together—can man live, still they are both foods, and if we take Bovril, or some of the jellies and extracts as examples of concentrated forms of certain nitrogenous elements, that is, as containing these elements and nothing else, true as it is that one cannot live on them they none the less are foods, capable of playing their proper part in building up a complete diet. All of this is, as we have said, a mere play upon words. Still, if there really are people who do in fact believe that an ox can be got into a teacup, Dr. Dickinson's remarks will go far to place the matter before them in a proper light.

